# Interacting hands: the role of attention for the joint Simon effect

**DOI:** 10.3389/fpsyg.2014.01462

**Published:** 2014-12-17

**Authors:** Roman Liepelt

**Affiliations:** Institute for Psychology, University of MuensterMuenster, Germany

**Keywords:** joint Simon effect, joint action, social interaction, joint hand posture effect, spatial attention, stimulus–response compatibility

## Abstract

Recent research in monkeys and humans has shown that the presence of the hands near an object enhances spatial processing for objects presented near the hand. This study aimed to test the effect of hand position on the joint Simon effect. In Experiment 1, two human co-actors shared a Simon task while placing their response hands either near the objects appearing on the monitor or away from the monitor. Experiment 2 varied each co-actor’s hand position independently. Experiment 3 tested whether enhanced spatial processing for objects presented near the hand is obtained when replacing one of the two co-actors by a non-human event-producing rubber hand. Experiment 1 provided evidence for a Simon effect. Hand position significantly modulated the size of the Simon effect in the joint Simon task showing an increased Simon effect when the hands of both actors were located near the objects on the monitor, than when they were located away from the monitor. Experiment 2 replicated this finding showing an increased Simon effect when the actor’s hand was located near the objects on the monitor, but only when the co-actor also produced action events in spatial reference. A similar hand position effect was observed in Experiment 3 when a non-human rubber hand replaced the human co-actor. These findings suggest that external action events that are produced in spatial reference bias the distribution of attention to the area near the hand. This strengthens the weight of the spatial response codes (referential coding) and hence increases the joint Simon effect.

## INTRODUCTION

Social context has an enormous impact on individual task performance. Observing another person’s action activates corresponding motor representations in the observer (Iacoboni et al., 1999), which may help when planning the same action or produces action conflict when planning or executing a different action (Brass et al., 2000; Liepelt et al., 2008, 2009; Sebanz and Knoblich, 2009; Wurm and Schubotz, 2012). Ideomotor theory (James, 1890; Prinz, 1997) and extensions thereof [theory of event coding, (TEC; Hommel et al., 2001)] assume that action control operates on the perceptual representations that usually follow a particular action. Action selection therefore consists in the activation of the perceptual consequences (i.e., action effects) of the to be generated action. Action perception, action selection and action execution use common representational codes leading to strong bidirectional interaction effects between perception and action (Prinz, 1990, 1997; Liepelt et al., 2011a). Such interactions also occur when we share tasks with others. Fluent interaction requires the coordination of both partners’ actions in space (Sebanz et al., 2003) and time (Sebanz et al., 2006; Liepelt and Prinz, 2011). Internal action plans need to be continuously updated according to ongoing changes in the arrangement of both actors’ bodies and body parts. Another person’s actions and body part positions represent an important information source to achieve fluent movement interactions between two actors.

Task sharing allows individuals to achieve common goals that they cannot achieve alone, which has played an important evolutionary role for group survival (Tomasello et al., 2005). Joint action research may therefore shed light into the most basic mechanisms on which more complex social cognitive functions, social institutions and societies are build on. Hand position plays an important role for joint action, because it can be considered to be a non-verbal cue to alert the co-actor’s attention to a particular location in space leading to a deeper processing of items appearing in near hand space. The present study examines the influence of relative hand position belonging to two different individuals on dyadic task sharing performance.

### DYADIC TASK SHARING AND THE JOINT SIMON EFFECT

An effective way to test dyadic task sharing effects is the joint Simon task (Sebanz et al., 2003). Here, two actors sit alongside each other sharing complementary parts of a standard Simon task (Simon and Wolf, 1963; Simon and Small, 1969; Simon et al., 1970). In the standard Simon task, a single person has to discriminate between two visual (e.g., different geometric forms) or two auditory (e.g., auditor words) target stimuli that are presented on the left or the right side of a screen or through the left and right speaker of a headphone. In most versions of the task, the participant had to respond exclusively to the shape of the visual stimulus or the meaning of the auditory stimulus by pressing a left or right button placed on the table in front of the person, but had to ignore the location (i.e., the stimulus position) where it was presented. Although stimulus position was fully task irrelevant, responses were typically faster when the stimulus position spatially corresponded with the response position [*Stimulus–Response (S–R) compatibility*], as when they did not correspond *(S–R incompatibility)* showing that participants were unable to ignore the spatial position of the stimulus (Simon effect). The Simon effect is often explained by assuming that response selection is facilitated in S–R compatible trials because the irrelevant stimulus features (left or right position of the stimulus) automatically prime the corresponding response. In S–R incompatible trials a response conflict arises between the automatically primed response and the instructed response (Kornblum et al., 1990; Hommel, 2011). Dimensional overlap is established between codes representing the irrelevant stimulus location and the features representing the response location (De Jong et al., 1994) or between the current or previous focus of attention to the left or right side following an attentional shift to the laterally presented stimuli and the spatially coded response (Nicoletti and Umiltá, 1989; Nicoletti, 1994). Note, however, that this mechanism is different from the Stroop effect (Stroop, 1935) or the Flanker effect (Eriksen and Eriksen, 1974) lacking a clear spatial S–R compatibility and a semantic conflict could arise between different stimulus features or between different response features that are mapped to multiple stimulus features (Hommel, 2011).

When one person has to respond to only one of the two stimuli (being in charge of only half of the task) with one button press (individual Simon task), the Simon effect usually disappears (Hommel, 1996), which makes sense as the alternative response location is removed eliminating the response conflict. When a second person takes over the other half of the task responding to the complementary stimulus, the Simon effect re-appears [joint Simon effect (JSE; Sebanz et al., 2003)], as in the standard Simon task. Sebanz and colleagues concluded that both participants sharing the Simon task form a common task representation corepresenting also the action of their co-actor (*action corepresentation*). Including both actors’ action alternatives in a common task representation re-establishes dimensional overlap between spatial stimulus and response dimensions. Just as in the standard Simon task this leads to response priming in S–R compatible trials and response conflict in S–R incompatible trials with the only difference that the two responses now relate to two different persons. Action corepresentation therefore supposes the processes underlying the joint Simon task and the standard Simon task to be functionally equivalent (Sebanz et al., 2003; Vesper et al., 2010).

The JSE is assumed to represent an index of action corepresentation (Sebanz et al., 2003) being sensitive to manipulations changing cognitive control parameters that regulate the integrativeness of control states thereby narrowing or broadening the attentional focus (Colzato et al., 2012, 2013). Further, manipulating the interaction quality between two humans (Hommel et al., 2009) and between humans and robots (Stenzel et al., 2012) has been shown to modulate the size of the JSE. Sharing a task with a friendly co-actor (Hommel et al., 2009) or a humanoid robot (Stenzel et al., 2012) that acted in a biologically inspired way increased the JSE, as compared to conditions in which the co-actor was unfriendly or the robot responded in a purely deterministic manner. The JSE seems to be disturbed in patients with frontal and parietal lesions incapable of performing Theory of Mind tasks (Humphreys and Bedford, 2011) and schizophrenia patients (Liepelt et al., 2012), but seem to be intact in patients with autism who pass first-order Theory of Mind tasks (Sebanz et al., 2005).

However, as the JSE represents a spatial compatibility effect, it strongly depends on the relative spatial setting between the irrelevant spatial stimulus information and the spatial response to be given (Sebanz et al., 2003), the spatial coding of responses (Dolk et al., 2011; Dittrich et al., 2012) and spatial attention to alternative events (e.g., a rhythm producing metronome) that provide a spatial reference frame for the coding of the participant’s own action as left or right (Dolk et al., 2013). Based on the latter findings a referential coding account has been proposed for the finding of a JSE, which is based on the general assumptions of ideomotor theory (James, 1890; Stock and Stock, 2004), and TEC (Hommel et al., 2001) in particular. TEC assumes that an individual controls his/her own intentional actions by the activation of feature codes that represent bundles of the perceivable effects this action typically produces. Accordingly, the perception of alternative action (or object) events representing the same or similar feature codes produces an action selection conflict between externally triggered and internally activated actions (i.e., their activated feature codes). The action selection conflict can be resolved by emphasizing (cf. intentional weighting principle; Memelink and Hommel, 2013) on action features that discriminate best between own and others actions—*referential coding* (Hommel, 1993; Hommel et al., 2001; Dolk et al., 2013; for a review see Dolk et al., 2014). According to the intentional weighting principle (Memelink and Hommel, 2013), the discrimination between two action events can be achieved by attending more strongly on response location changing the weight of the spatial (left–right) location code of each individual actor’s actions (Dolk et al., 2011, 2013). Spatial coding of the participant’s own response again introduces dimensional feature overlap between lateralized stimuli and both actors’ responses (Kornblum et al., 1990) and hence might explain the re-appearance of the Simon effect in a joint-task setting without needing the assumption of task corepresentation (Liepelt et al., 2011b). In the Simon task the best discriminating features are spatial response features. However, according to the referential coding account, in principle all other features could be used for referential coding depending on the specific task that two people share and the level at which the action selection conflict arises (Sellaro et al., in press). One should note here that referential coding might also include features that are often considered to be social (e.g., intentionality, in-group vs. out-group membership, agency, etc.), which can also be used to discriminate between actions produced by different actors (Dolk et al., 2014).

The JSE is typically not observed when the spatial reference that is usually provided by a human co-actor is made less salient (Guagnano et al., 2010; but see also Welsh et al., 2013). Guagnano et al. (2010) showed that the JSE, which is observed when two actors respond in peripersonal space, is diminished when they are located in extrapersonal space (out of reach distance). A similar finding is observed when the co-actor is placed in a separate room, which is spatially not further specified (Welsh et al., 2007; Sellaro et al., 2013; but see Tsai et al., 2008).

### ENHANCED PROCESSING OF STIMULI NEAR THE HAND

Recent research provided evidence for effects of action on perception by showing how hands alter visual processing (Schendel and Robertson, 2004). Schendel and Robertson (2004) tested a patient with a damage of the right primary visual cortex who had a considerable loss in the left visual hemifield in a visual detection task. They showed that the patient was able to significantly improve the detection rate in their damaged visual field when reaching with their left-hand out to the left side of the monitor as when placing the hand to his lap. These findings are consistent with the finding of bimodal (visual and tactile) neurons in the putamen (Graziano and Gross, 1995), the premotor cortex (Graziano et al., 1994) and the parietal cortex (Colby et al., 1993; Iriki et al., 1996) of monkeys coding the space around the body (i.e., the monkey’s hand). Graziano and Gross (1995) showed that receptive fields of these neurons have a specific characteristic. They move with current hand position (Graziano and Gross, 1995) providing the neural substrate for altered vision near the hands (Graziano and Gross, 1995; Maravita and Iriki, 2004).

By using functional magnetic resonance imaging, brain areas have also been found in the human intraparietal sulcus that represent multisensory information in a hand-centered space (Makin et al., 2007). Cognitive research provides evidence for the role of attention for the finding of altered vision near the hands (Reed et al., 2006, 2010; Abrams et al., 2008; Witt and Proffitt, 2008; Davoli and Abrams, 2009; Davoli et al., 2010, 2012; Tseng and Bridgeman, 2011; Davoli and Brockmole, 2012; Qian et al., 2012; Schultheis and Carlson, 2013; Abrams and Weidler, 2014; Weidler and Abrams, 2014). Taking things in our hands not only affects visual search (Abrams et al., 2008), but has also profound effects on perception, attention and memory (Brockmole et al., 2013). Most studies testing effects of hand position on task performance compared performance differences between two conditions. One in which the participant responded with two response buttons attached to the display (objects close to the hands) and the other in which the person responded with both buttons located on participant’s knees (objects away from the hands). But why are objects close to the hands represented differently from objects away from the hands? A reasonable explanation given is that objects close to the hands are possible candidates for action. Since we know from ideomotor theory (James, 1890), and TEC (Hommel et al., 2001) that action and perception are tightly linked together, it makes sense to assume that the presence of the hand near an object can change the way in which we process that object (i.e., the way the object is represented). Most of the given evidence suggests that the presence of the hands enhances spatial processing for (or biases the distribution of attention to) the area near the hands (Schendel and Robertson, 2004; Reed et al., 2006; Abrams et al., 2008; Davoli et al., 2010; for a review see Brockmole et al., 2013). However, recent studies found evidence for reduced Stroop interference (Davoli et al., 2010) and reduced task-switching costs (Weidler and Abrams, 2014) for near hand stimuli providing evidence for enhanced cognitive control for stimuli that appear near the hands.

As the joint Simon task is a tool to investigate spatial processing also involving cognitive control processes, and if we assume that the position of our hands change the salience of space by providing references for upcoming actions (Dolk et al., 2013), then hand position should have a strong impact on the JSE. The aim of the present study was to systematically test the effect of hand position on the JSE.

## EXPERIMENT 1

Participants had to perform three types of Simon tasks (individual Simon task, joint Simon task, and standard Simon task). Stimuli were geometric figures (square and diamond) that randomly appeared on the left or the right side of a centrally presented fixation cross. In the standard Simon task, participants were asked to press a left key for square and a right key for diamond. In both, the individual and the joint Simon task, participants responded to only one of the shapes by making a simple discrimination response. They were asked to refrain from responding when the other shape appeared.

Additionally, hand position was manipulated (Reed et al., 2006; Abrams et al., 2008). Two co-acting participants had to respond with buttons that were either located near the stimuli (hands monitor condition) or far away from the stimuli with their response buttons placed on their knees (hands knee condition). This manipulation was applied to all three tasks (individual Simon, joint Simon and standard Simon task). While the hand location was related to both hands of an individual actor in the standard Simon task, it was related to two different actors in the joint Simon task.

If hand posture biases the distribution of attention to the space near one’s own hand, one should predict a larger JSE when both actors’ response hands are placed on the monitor as when they are located on their knees. However, if people exhibit enhanced cognitive control for stimuli that appear near the hands (Weidler and Abrams, 2014), one should predict a smaller JSE when both actors’ responses are placed on the monitor as when they are located on their knees.

### METHODS

#### Participants

A sample of 24 students (12 male; mean age, 24.6 years; SD = 2.2) participated in this experiment. All were right-handed, had normal or corrected-to-normal vision, and were naive with regard to the hypotheses of the experiment. They were paid €7 or course credit points for taking part in the study. Participants gave their informed consent to participate in the study, which was conducted in accordance with the ethical standards laid down in the 1975 Declaration of Helsinki.

#### Stimuli and apparatus

**FIGURE 1:**
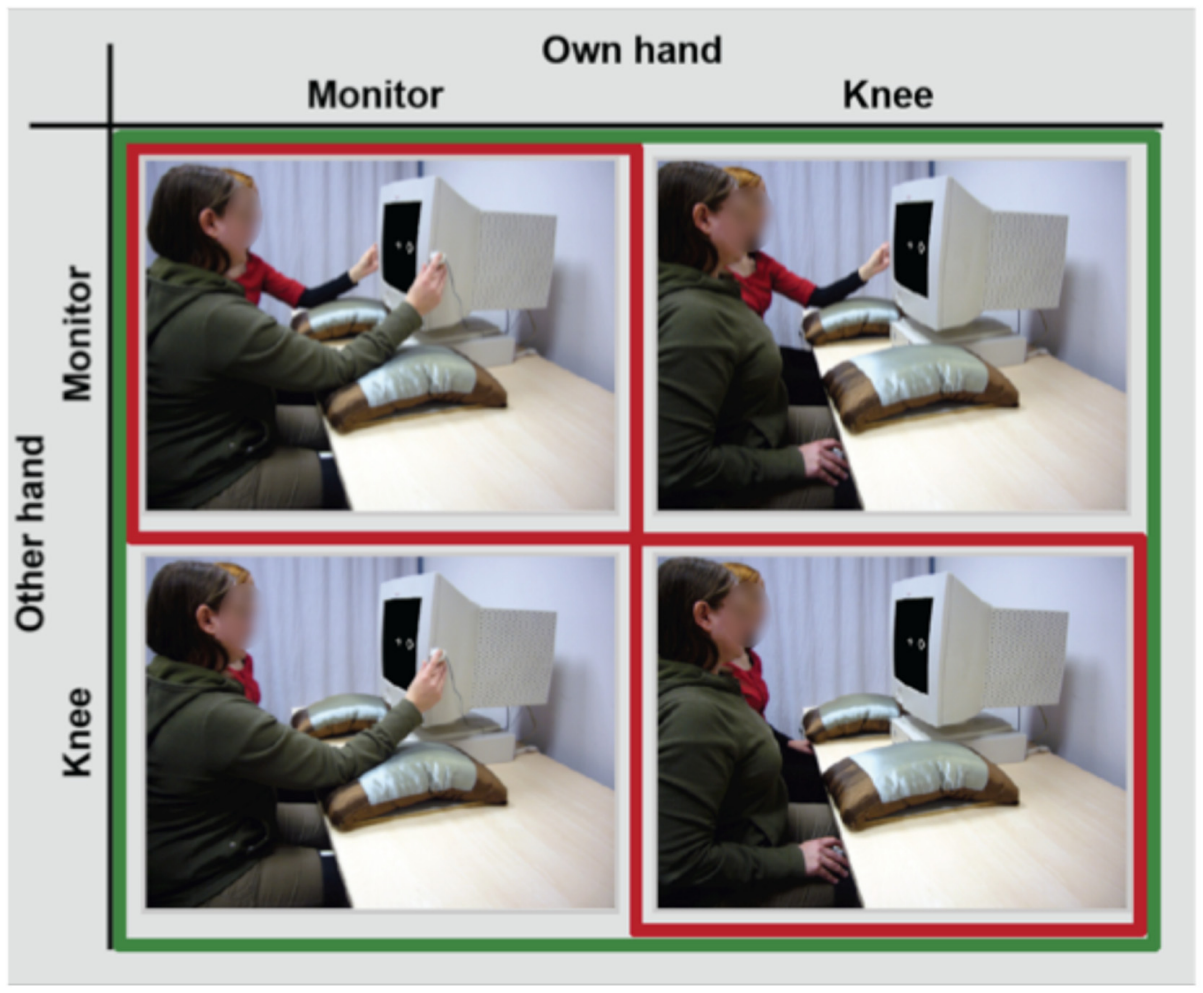
**Experimental design of the joint Simon task of Experiment 1 (red border) showing two persons sharing a visual Simon task (own hand: right person and other hand: left person) in the hands knee condition (lower right panel) and the hands monitor condition (upper left panel).** In Experiment 1, all participants also performed an individual and a standard version of the Simon task using the same hand position manipulation (not shown). Experimental design for the joint Simon task of Experiment 2 (green border) showing the own hands knee – other hands monitor condition (upper right panel) and the own hands monitor – other hands knee condition (lower left panel) in addition.

Participants were seated in a sound-attenuated, dimly lit room. All stimuli were displayed on a CRT computer monitor (19-inch) in white on a black background at a constant viewing distance of 60 cm. The fixation point at the center of the screen was marked by a plus sign (0.9 × 0.9°). Stimuli consisted of squares and diamonds (1.9 × 1.9°), presented to the left or right of the fixation cross with an eccentricity of 5.7° visual angle. Responses were recorded with two keys. The keys were either placed on participants’ left or right knee or at the left and right side of the computer monitor with a distance of 50 cm from each other (see **Figure 1**, red border). The distance between response keys in the hands monitor and the hands knee condition was approximately kept constant. This was achieved by placing the response buttons on the outer knees that were matched in distance to the response buttons in the hands monitor condition.

#### Task and procedure

In the standard Simon task, participants responded by pressing the left key with the index finger of their left-hand for the square and the right key with their right index finger for the diamond. The stimuli randomly appeared on the left or the right of the centrally presented fixation cross. Participants were seated to either the left or the right side in front of the monitor in the joint Simon task. To keep the seating position identical in all three tasks an empty chair remained in place in the individual Simon task and the standard Simon task.

In the individual and the joint Simon tasks, participants responded to one of the shapes only (e.g., squares) by making a simple discrimination response. They were asked to refrain from responding if the other shape (e.g., diamond) appeared. In the joint Simon task, they performed the identical task sitting alongside another person who responded to the other stimulus. The left-sitting person responded with the left hand and the right-sitting person with the right-hand. In the standard Simon task left-hand and right-hand responses were related to a single individual.

Each trial began with the presentation of a fixation cross for 250 ms. The target stimulus (square or diamond) appeared together with the fixation cross for 150 ms. Afterward, responses had to be given within 1800 ms. In the case of correct responses, the fixation cross was provided as feedback for 300 ms. If no response was given within 1800 ms after stimulus onset, the feedback “zu langsam” (too slow) was shown. In the case of an incorrect response, error feedback “Fehler” (error) was provided. All forms of feedback (fixation cross, too slow, or error) were displayed for 300 ms. Following feedback, there was a constant inter-trial interval of 1750 ms before the next trial started. In each task, participants completed five experimental blocks of 112 trials, separated by short breaks. Before each task, participants performed a block of 66 practice trials.

Each participant performed the experiment on two consecutive days, performing all three tasks with a different hand position on each day. Half of the participants started with the hands monitor condition, the other half with the hands knee condition. The order of hand positions and tasks were counter-balanced across pairs of participants.

### RESULTS

For statistical reaction time (RT) analysis, all trials in which responses were incorrect (2.2%), and RTs were faster than 150 ms or slower than 900 ms (1%) were excluded to avoid distortions of statistic estimates. To test effects of hand position on the Simon task a 3 × 2 × 2 repeated measures analyses of variance (ANOVA) with the within-subjects factors setting (individual Simon task, joint Simon task, standard Simon task), hand position (hands monitor, hands knee) and compatibility (compatible, incompatible) was performed separately for RTs and percent errors. Planned *post hoc* comparisons were performed when required.

#### Reaction times

**FIGURE 2:**
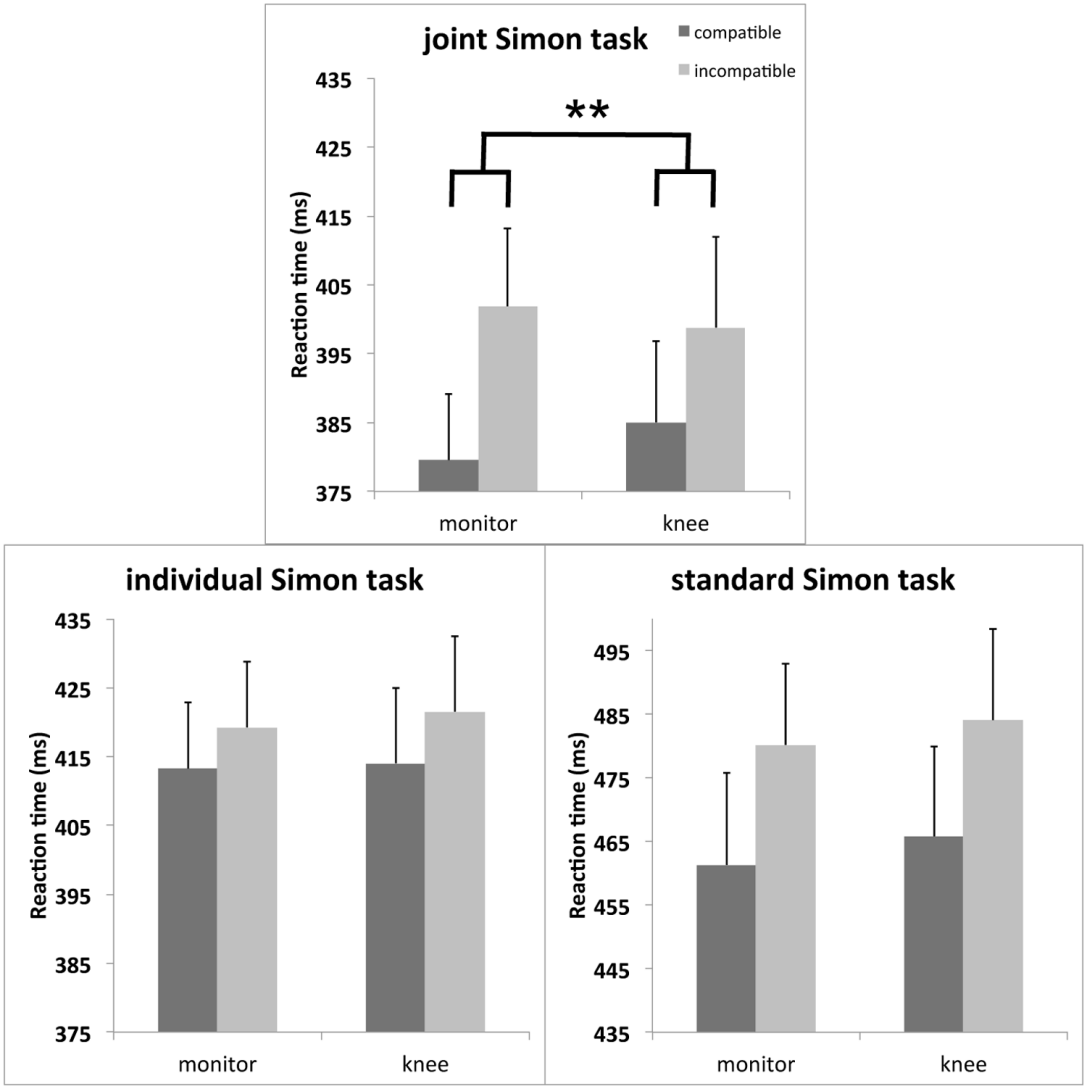
Mean reaction time (RT; in milliseconds) of the joint Simon task (upper middle panel), the individual Simon task (lower left panel) and the standard Simon task (lower right panel) as a function of hand position (hands monitor, hands knee) and compatibility (compatible, incompatible) of Experiment 1 (**p < 0.01 indicating the interaction of hand position and compatibility in the joint Simon task). Error bars depict the SE of the mean.

The RT analyses showed a significant main effect of compatibility, *F*(1,23) = 61.42, *p* < 0.001, partial η^2^ = 0.73, which indicated that irrespective of setting and hand position, responses were faster with S–R compatibility (420 ms) than with incompatibility (434 ms), reflecting an overall Simon effect. The analysis also revealed a significant main effect of setting, *F*(2,46) = 44.06, *p* < 0.001, partial η^2^ = 0.66. RTs in the joint Simon task were faster (391 ms) as compared to the individual Simon task (417 ms) and the standard Simon task (473 ms). Both factors, setting and compatibility significantly interacted, *F*(2,46) = 9.01, *p* < 0.001, partial η^2^ = 0.28, showing that the compatibility effects was significantly increased in the joint Simon task (18 ms, *p* < 0.001), as compared to the individual Simon task (7 ms, *p* = 0.001), *F*(1,23) = 19.26, *p* < 0.001, partial η^2^ = 0.46, while the compatibility effects between the joint Simon task and the standard Simon task (19 ms, *p* < 0.001) were not statistically different (*F* < 1). Further, there was a significant three-way interaction of setting, hand position and compatibility, *F*(2,46) = 3.37, *p* = 0.043, partial η^2^ = 0.13 (see **Figure 2**) showing that hand position had a stronger impact on the compatibility effect in the joint Simon task, as in the individual Simon task, *F*(1,23) = 6.44, *p* = 0.018, partial η^2^ = 0.22, and the standard Simon task *F*(1,23) = 4.20, *p* = 0.05, partial η^2^ = 0.15 ^1^. The significant interaction of hand position × compatibility in the joint Simon task, *F*(1,23) = 9.64, *p* = 0.005, partial η^2^ = 0.30, indicated a larger compatibility effect when both actors’ hands were located on the monitor (22 ms, *t* = 7.1, *p* < 0.001), as when they were located on their knee (14 ms, *t* = 4.8, *p* < 0.001). No such effect of hand position on the compatibility effect was observed in the individual and the standard Simon task (both *F*s < 1). No further main effects or interactions reached statistical significance (all *ps* > 0.05).

#### Error rates

For errors (see **Table 1**), there was a significant main effect of compatibility (1.8% in compatible trials and 2.6% in incompatible trials), *F*(1,23) = 16.89, *p* < 0.001, partial η^2^ = 0.42, and a main effect of setting, *F*(2,46) = 29.56, *p* < 0.001, partial η^2^ = 0.56. Also the interaction of setting and compatibility was significant, *F*(2,46) = 3.45, *p* = 0.040, partial η^2^ = 0.13. We observed significant compatibility effects in the standard Simon task (3.1% in compatible trials and 4.8% in incompatible trials, *p* = 0.011) and in the joint Simon task (1.6% in compatible trials and 2.3% in incompatible trials, *p* = 0.044), but not in the individual Simon task (*F* < 1). No further main effects or interactions reached statistical significance (all *ps* > 0.05).

**Table 1:** Error rates (percentage) shown for different Simon task settings (joint, standard, and individual) as a function of other’s hand position (other hand monitor, other hand knee), participant’s hand position (own hand monitor, own hand knee), and compatibility (C, compatible; IC, incompatible) for Experiment 1.

	Other hand monitor				Other hand knee			
	Own hand monitor		Own hand knee		Own hand monitor		Own hand knee	
	C	IC	C	IC	C	IC	C	IC
Joint	1.4	2.6	–	–	–	–	1.8	2.1
Standard	3.1	4,5	–	–	–	–	3.0	4.6
Individual	0.6	0.7	–	–	–	–	0.8	0.8

### DISCUSSION

Experiment 1 showed a JSE in the joint Simon task and the standard Simon task, as well as a tiny effect in the individual Simon task. Hand posture modulated the size of the Simon effect only during joint-task processing. When the hands of both actors were located near the monitor, the JSE was significantly larger, as when both actors’ response hands were more distant to the monitor. These data are in line with the prediction that an attention capturing hand of the co-actor located near the monitor biases the distribution of attention to the space near one’s own hand in the joint Simon task. Using a distribution analyses, it could be ruled out that the hand posture effect found in the joint Simon task was not due to a social facilitation effect and faster RTs in the joint Simon task as compared to the individual Simon task and the standard Simon task. The error data support the RT findings but are as in most studies measuring the JSE less meaningful, which is probably due to the relatively low overall error rate.

The finding of a Simon effect in the joint Simon task and of a tiny effect in the individual Simon task is in line with recent findings of other studies (Hommel, 1996; Liepelt et al., 2011b; Dolk et al., 2013) suggesting that the Simon effect is the result of an action discrimination problem between internally and externally triggered actions and its corresponding resolution by referential coding (Dolk et al., 2013, 2014). The hand position on the monitor seems to enhance the attention to the spatial response location leading to a larger JSE. This conclusion is in line with the finding of a relatively large JSE in the present study, as compared to previous studies measuring the JSE (Sebanz et al., 2003; Liepelt et al., 2011b, 2013; Dolk et al., 2013) using the standard response key arrangement and response hands placed on the table.

## EXPERIMENT 2

In the first experiment both actors always moved their hands together either to the monitor or the knee position. Experiment 2 aimed to isolate effects of own and other’s hand positions, as well as their potential interactions, using the joint Simon task of Experiment 1. This experiment applied four different hand position conditions. While again using the two hand position conditions of Experiment 1 (own hand monitor–other hand monitor, own hand knee–other hand knee) to test for a replication of the main findings of Experiment 1, two new conditions were added (own hand monitor–other hand knee, other hand monitor–own hand knee). These two new conditions allow separating effects of one’s own hand position from effects of the co-actor’s hand position. If own hand position produced the spatial attentional bias that was found in Experiment 1, one should predict a larger JSE when the actor’s own response hand is placed near the monitor, as when it is placed away from the monitor. However, when the other person’s hand position modulated attention, one should find a larger JSE when the other actor’s response is placed near the monitor, as when it is placed away from the monitor. Finally, biasing the distribution of attention to one’s own task space may interact with intentional weighting (Dolk et al., 2013) so that a stronger weighting (of discriminative action features) can compensate effects of enhanced spatial attention. In this case, enhanced spatial processing of the stimuli in near hand space should affect the JSE when the other person’s hand is also located at the monitor, but less when the other person’s hand is located on the knee.

### METHOD

#### Participants

A new sample of 32 students (eight male; mean age, 25.9 years; SD = 5.8) participated in this experiment and fulfilled the same criteria as participants in Experiment 1.

#### Stimuli and apparatus

Stimuli and apparatus were the same as in the joint Simon task of Experiment 1.

#### Task and procedure

Procedure and design were identical to the joint Simon task of Experiment 1, except that two new hand position conditions were added (see **Figure 1**, green border), in order to separately manipulate the hand positions of actor (own hand monitor, own hand knee) and co-actor (other hand monitor, other hand knee). All hand position conditions were counter-balanced across pairs of participants.

### RESULTS

Using the same outlier criterion as in Experiment 1 led to the exclusion of incorrect trials (2.7%) and RT outliers (0.5%). To test effects of hand position on the joint Simon task a 2 × 2 × 2 repeated measures ANOVA with the within-subjects factors own hand position (hands monitor, hands knee), other hand position (hands monitor, hands knee) and compatibility (compatible, incompatible) was performed separately for RTs and percent errors. Planned *post hoc* comparisons were performed when required.

#### Reaction times

**FIGURE 3:**
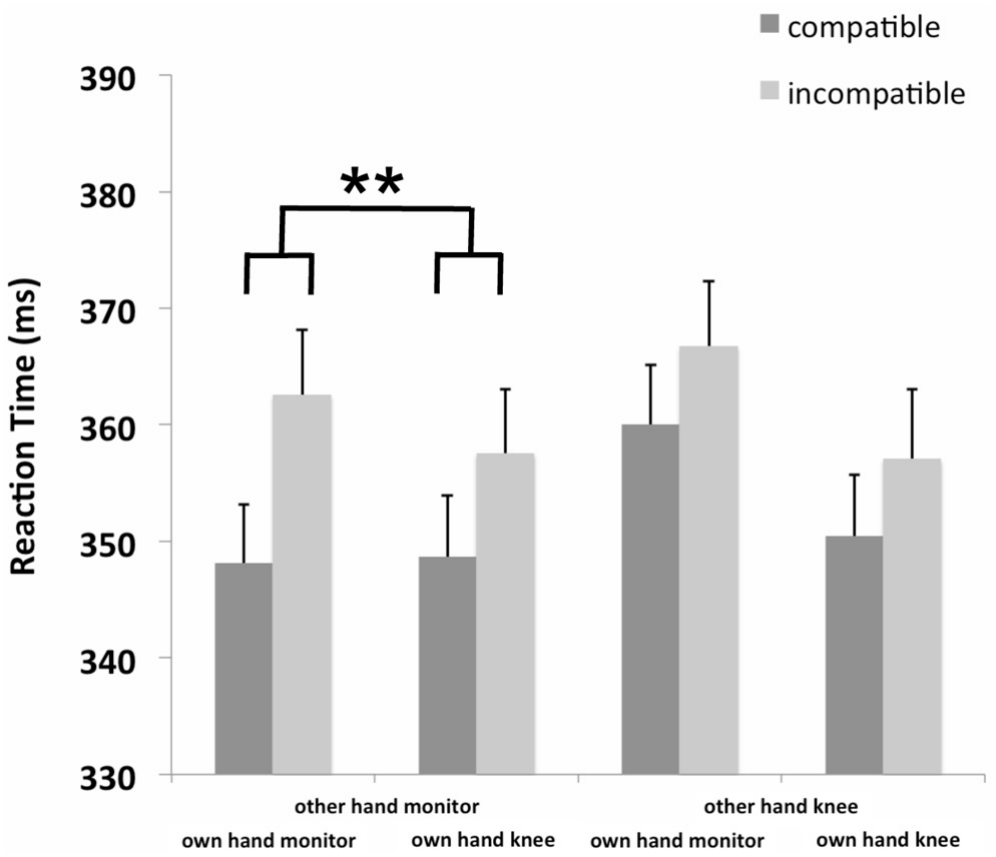
Mean RT (in milliseconds) of the joint Simon task as a function of other hand position (hands monitor, hands knee), own hand position (hands monitor, hands knee) and compatibility (compatible, incompatible) of Experiment 2 (**p < 0.01 indicating the interaction of hand position and compatibility). Error bars depict the SE of the mean.

The RT analyses (see **Figure 3**) showed a significant main effect of own hand position, *F*(1,31) = 5.54, *p* = 0.025, partial η^2^= 0.15 with slower RTs for the own hand monitor (359 ms), as for the own hand knee condition (353 ms). We also found a significant interaction of own hand position × other hand position, *F*(1,31) = 5.29, *p* = 0.028, partial η^2^ = 0.15 showing that the RT difference between the own hand monitor (363 ms) and the own hand knee condition (354 ms) present for the other hand knee condition was decreased when the other hand was located on the monitor (355 ms vs. 353 ms, respectively). The main effect of compatibility was also significant, *F*(1,31) = 31.49, *p* < 0.001, partial η^2^ = 0.50, indicating that irrespective of hand position, responses were faster with S–R compatibility (352 ms) than with incompatibility (361 ms), showing an overall JSE amounting to 9 ms.

Most importantly, the actor’s own hand position, the co-actor’s hand position, and compatibility interacted significantly, *F*(1,31) = 6.72, *p* = 0.014, partial η^2^ = 0.18, showing that the JSE was larger for the own hand monitor position (14 ms), as for the own hand knee position (9 ms) when the co-actor’s hand was located on the monitor, *F*(1,31) = 9.28, *p* = 0.005, partial η^2^ = 0.23, but not when the co-actor’s hand was located on the knee (7 ms vs. 7 ms, respectively, *F* < 1; see **Figure 3**). The increased JSE for the own hand monitor position when the co-actor also responded on the monitor is mainly driven by a difference on incompatible trials (*p* = 0.049, one-tailed). The relatively large compatibility effect when actor’s and co-actor’s hands were positioned at the monitor, indicated by the significant three-way interaction, also explains the significant two-way interactions of own hand position x compatibility, *F*(1,31) = 4.24, *p* = 0.048, partial η^2^ = 0.12, showing an enlarged compatibility effect for the own hand monitor position (11 ms) than for the own hand knee condition (8 ms) and of other hand position × compatibility, *F*(1,31) = 17.38, *p* < 0.001, partial η^2^ = 0.36, showing an enlarged compatibility effect for the other hand monitor position (12 ms) than for the other hand knee condition (7 ms). No further main effects or interactions reached statistical significance (all *ps* > 0.05).

For reasons of completeness, the finding of a larger JSE for own hand monitor–other hand monitor, as compared to own hand knee–other hand knee conditions is also reported, *F*(1,31) = 15.14, *p* < 0.001, partial η^2^ = 0.33, showing a replication of the findings of Experiment 1.

#### Error rates

For errors (see **Table 2**), there was a significant main effect of compatibility (2.0% in compatible trials and 3.4% in incompatible trials, *F*(1,31) = 27.89, *p* < 0.001, partial η^2^ = 0.47, as well as a significant interaction of own hand position × other hand position, *F*(1,31) = 4.61, *p* = 0.040, partial η^2^ = 0.013, showing more errors for the own hand monitor position (3.0%) than for the own hand knee position (2.4%) when the co-actor’s hand was located on the monitor, while this was not the case when the co-actor’s hand was located on the knee (2.5% vs. 2.8%, respectively). No further main effects or interactions reached statistical significance (all *ps* > 0.05).

**Table 2:** Error rates (percentage) shown for the joint Simon task (joint) as a function of other’s hand position (other hand monitor, other hand knee), participant’s hand position (own hand monitor, own hand knee) and compatibility (C, compatible; IC, incompatible) for Experiment 2.

	Other hand monitor				Other hand knee			
	Own hand monitor		Own hand knee		Own hand monitor		Own hand knee	
	C	IC	C	IC	C	IC	C	IC
Joint	2.1	3.9	1.7	3.1	2.0	3.1	2.3	3.4

### DISCUSSION

Using a joint Simon task Experiment 2 tested if the enhancement of spatial attention to the space near one’s own hand depends on the other person’s hand position. Overall, this experiment provides evidence for a JSE, replicating previous findings (Sebanz et al., 2003; Liepelt et al., 2011b). The hand position of both actors modulated the size of the JSE. Experiment 2 replicated the effect of hand position found in Experiment 1, showing a larger JSE when both actors’ responses were located near the monitor, as when placing their hands on their knees. The JSE was enlarged when the actor’s own hand was positioned on the monitor, as compared to a condition where it was located on the knee, but only when the co-actor’s hand was also located on the monitor. The enhancement of spatial processing in the area near the own hand seems to increase the response selection conflict between the automatically primed and the instructed response (Kornblum et al., 1990) when the stimulus and the response do not correspond which is compensated by intentional weighting (Memelink and Hommel, 2013).

## EXPERIMENT 3

Based on the finding of a JSE when two human co-actors jointly perform a Simon task, Sebanz et al. (2003) proposed that two persons sharing a (Simon) task form a common task representation—corepresenting the action of their partner (*action corepresentation*). Indeed there is evidence showing that the JSE is increased when interacting with an intentional agent than with a non-intentional agent (Tsai and Brass, 2007; Müller et al., 2011; Stenzel et al., 2012) and when agency of the co-actor can be perceived (Stenzel et al., 2014). However, based on the finding that a JSE is not only observed for human co-actors, but can also be induced for non-human event-producing objects, Dolk et al. (2011, 2013) proposed an alternative account for the finding of the JSE—the referential coding account. The referential coding account cannot only explain the finding of a JSE in human and non-human co-actors, it also provides a comprehensive explanation why the JSE is increased when interacting with a friendly than with an unfriendly co-actor (Hommel et al., 2009), with individuals that were primed to a divergent thinking style (Colzato et al., 2013) or when perceiving agency (Stenzel et al., 2014; for a review see Dolk et al., 2014). The core assumption the referential coding account holds is that the discrimination between two (action) events can be achieved by intentional weighting of discriminative action features (Memelink and Hommel, 2013), which in the case of the Simon task are spatial features (left–right location codes).

The present study shows how attention to near hand space and intentional weighting may interact. When this is true, one should also find evidence for an attentional bias when the human co-actor is replaced by an event-producing non-human co-actor (e.g., a rubber hand). Experiment 3 aims to test if the actor’s hand position on the monitor biases the distribution of attention to the space near the hand when a non-living rubber hand produces (action) events on the monitor.

If biasing the distribution of attention to one’s own task space increases the action selection conflict when perceiving alternative (action) events in spatial reference and hence the need for a stronger weighting (of discriminative action features), one should find the own hand space bias when an event-producing rubber hand is located at the monitor, but fewer when the rubber hand is located away from the monitor.

### METHOD

#### Participants

A new sample of 32 students (11 male; mean age, 23.8 years; SD = 3.9) participated in this experiment. One participant had to be excluded due to a technical problem during data recording. Participants fulfilled the same criteria as participants in the previous experiments.

#### Stimuli and apparatus

**FIGURE 4:**
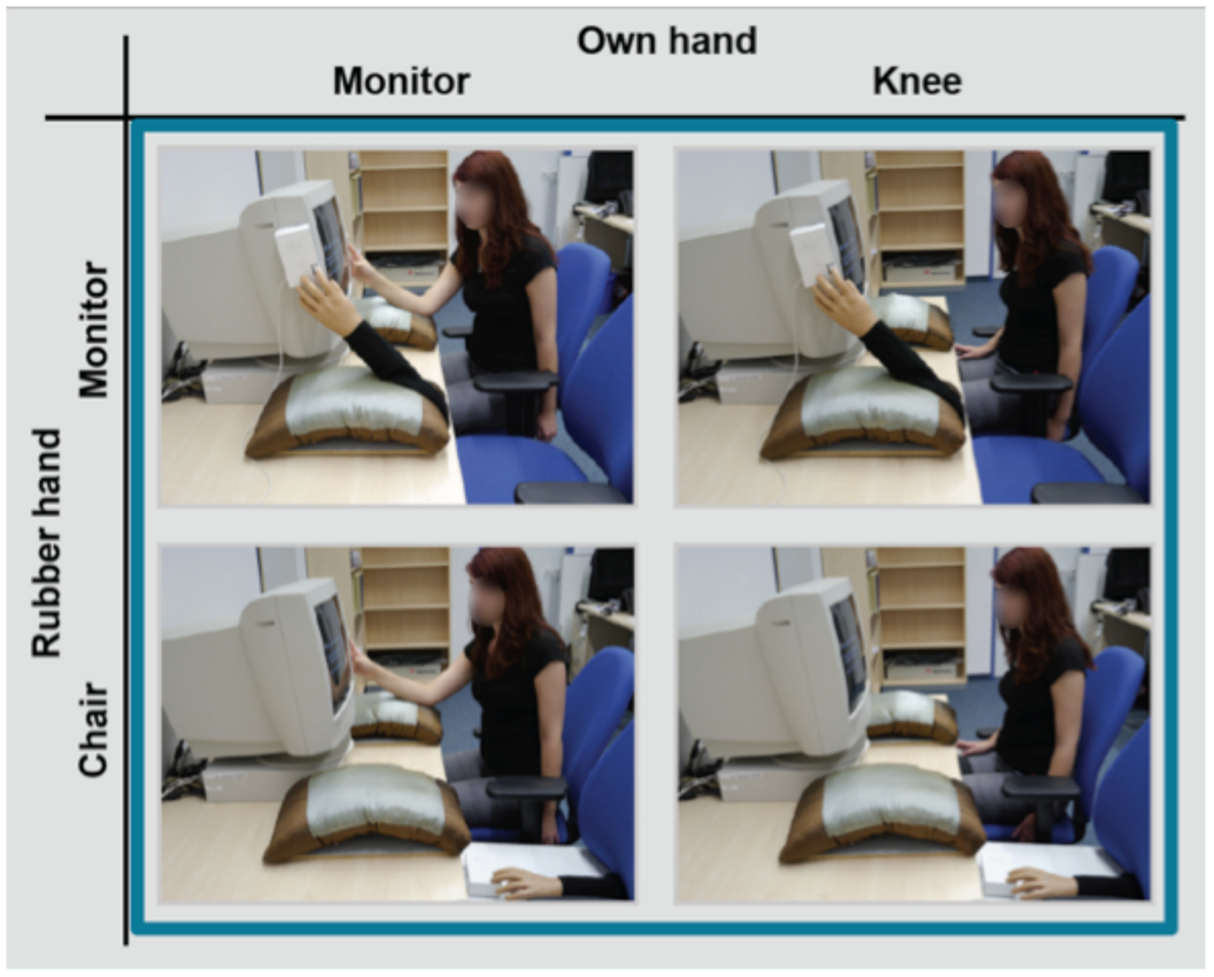
**Experimental design of the joint Simon task of Experiment 3.** The same hand position manipulation was used as in Experiment 2, but in Experiment 3, the left human co-actor was replaced by a rubber hand positioned on a response button, which pulled down the index finger of the rubber hand when the square appeared. The figure shows the hands monitor and the hands knee condition for the participant’s own hand and hands monitor and the hands chair condition for the rubber hand.

Stimuli and apparatus were the same as in Experiment 2 with the exception that a response device, which was triggered by the computer, replaced the conventional response button on the left side (hands monitor and hands knee conditions). The response device pulled down the index finger of a rubber hand whenever the square appeared on the screen, as in the typical joint Simon task (see **Figure 4**).

#### Task and procedure

Procedure and design were identical to Experiment 2, with the exception that in the joint Simon task of Experiment 3 the human co-actor sitting on the left side was replaced by a non-human agent (i.e., a rubber hand) that was attached to a computer triggered response device. The hand position conditions of own hand and rubber hand (see **Figure 4**) were counter-balanced across participants.

### RESULTS

Using the same outlier criterion as in the previous experiments led to the exclusion of 1.3% incorrect responses and 0.07% RT outliers. Data were analyzed as in Experiment 2.

#### Reaction times

**FIGURE 5:**
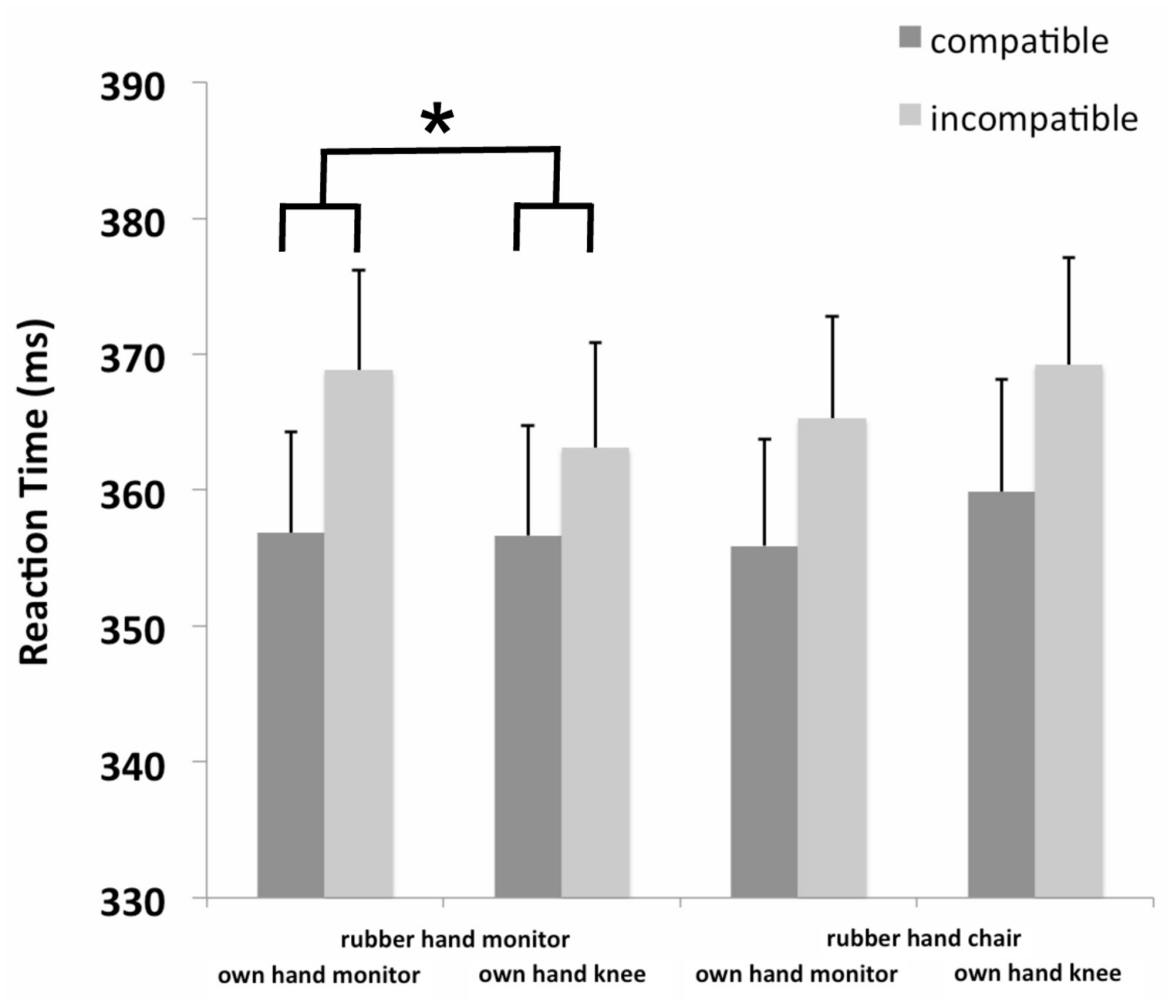
Mean RT (in ms) of the joint agent Simon task as a function of rubber hand position (hands monitor, hands chair), own hand position (hands monitor, hands knee) and compatibility (compatible, incompatible) of Experiment 3 (*p < 0.05 indicating the interaction of hand position and compatibility). Error bars depict the SE of the mean.

The RT analyses (see **Figure 5**) showed a significant main effect of compatibility, *F*(1,30) = 48.88, *p* < 0.001, partial η^2^ = 0.62, indicating that responses were overall faster with S–R compatibility (357 ms) than with incompatibility (366 ms), providing evidence for an overall JSE amounting to 9 ms. The co-agent’s hand position, the actor’s own hand position and compatibility interacted significantly, *F*(1,30) = 4.86, *p* = 0.035, partial η^2^ = 0.14. This three-way interaction shows that the compatibility effect was larger in the own hand monitor position (12 ms), than in the own hand knee position (6 ms) when the rubber hand was located on the monitor, *F*(1,30) = 6.06, *p* = 0.020, partial η^2^ = 0.17, but not when the rubber hand was located on the chair (9 ms vs. 9 ms, respectively, *F* < 1; see **Figure 5**). The increased JSE for the own hand monitor position when the rubber hand also responded on the monitor was mainly driven by a difference on incompatible trials (*p* = 0.033, one-tailed). No further main effects or interactions reached statistical significance (all *ps* > 0.05).

#### Error rates

For errors there were no significant (*p* < 0.05) main effects or interactions, which was probably due to overall low error rates (see **Table 3**).

**Table 3:** Error rates (percentage) shown for the joint agent Simon task (joint) as a function of rubber hand position (rubber hand monitor, rubber hand chair), participant’s hand position (own hand monitor, own hand knee) and compatibility (C, compatible, IC, incompatible) for Experiment 3.

	Rubber hand monitor				Rubber hand chair			
	Own hand monitor		Own hand knee		Own hand monitor		Own hand knee	
	C	IC	C	IC	C	IC	C	IC
Joint	1.4	1.1	1.3	1.5	1.3	1.4	1.5	1.4

### DISCUSSION

The findings of Experiment 3 are in line with the assumption that biasing the distribution of attention to one’s own task space leads to an increased action selection conflict when perceiving alternative (action) events in spatial reference. This action selection conflict can be resolved by a stronger weighting of discriminative action features—referential coding (Dolk et al., 2013, 2014). A finding that supports this conclusion is that the increased JSE when actor and rubber hand respond on the monitor is mainly driven by a difference on incompatible trials (i.e., an increased response selection conflict under S–R non-correspondence).

## GENERAL DISCUSSION

Previous research provides evidence for enhanced spatial processing of the area near the hands showing a strong coupling between action and perception. As the joint Simon task represents a central tool to investigate the role of spatial processing during joint action, the present study tested the role of hand position belonging to two different individuals in the joint Simon task.

### ENHANCED PROCESSING OF STIMULI NEAR THE HAND IN A DYADIC TASK-SHARING SITUATION

Experiment 1 showed that hand posture did significantly modulate the size of the Simon effect in the joint Simon task. The JSE was significantly larger when the hands of both co-actors sharing the Simon task were located near the monitor, as when they were located away from the monitor. Given that the hand position of both actors on the monitor changes the salience of space by providing references for upcoming actions (Dolk et al., 2013), these findings seem to be in line with the assumption that hand posture biases the distribution of attention to the space near one’s own hand (Reed et al., 2006) in the joint Simon task.

There was neither an effect of hand position on the Simon effect in the standard Simon task, nor in the individual Simon task. Therefore, these data are not yet decisive regarding the discussion of enhanced spatial processing (Reed et al., 2006; Abrams et al., 2008) versus enhanced cognitive control for items near the hand (Weidler and Abrams, 2014) for the standard Simon task. The findings in the standard Simon task and the individual Simon task may appear puzzling at first, but become clearer when considering the findings of Experiment 2. Experiment 2 replicated the hand position effect of Experiment 1, showing a larger Simon effect when both actors’ hands were located near the monitor, as when they were placed in a more distant hand position. This effect was only present for an actor when the co-actor also responded on the monitor, but not when responding with the hand away from the monitor. As participant’s RTs were faster in the joint Simon task, as compared to the individual and the standard Simon task, the enhancement of spatial processing in the joint Simon task may have been driven by participant’s motivation or perceived competition, which could have reduced variability in the data. However, with the findings of the distribution analyses this alternative could be ruled out showing that the hand posture effect in the joint Simon task was not due to differences in response speed.

The given findings suggests that enhanced spatial processing, which is a mechanism that has previously been shown in individual task settings (Reed et al., 2006; Abrams et al., 2008) seems to also affect joint-task processing when others produce (action) events in near task space. In the individual Simon task there is no other hand responding in spatial reference and hence no increased action selection conflict, which may explain why there was no effect of hand posture on the Simon effect in the individual Simon task. I can only speculate why there was no effect of hand position on the standard Simon task in the present study. One potential explanation might be that participants had quite a lot of practice (i.e., 560 trials in each task) with the Simon task in Experiment 1, as all types of Simon tasks were given in a counterbalanced order to participants. However, participants had an equal amount of practice in the joint Simon task and the standard Simon task. One can therefore conclude that the hand position effect observed in the joint Simon task seems to be more robust than a potential hand position effect in the standard Simon task. This conclusion is also confirmed by the findings of significant, but somewhat smaller hand posture effects on the JSE found in Experiments 2 and 3 when applying four different hand position conditions. A recent study of Schultheis and Carlson (2013) has shown that a larger number of hand postures extinguished the hand position effect in an individual task setting using the visual search task. The larger number of hand postures used in Experiment 2 and 3 (four postures in each experiment), as compared to Experiment 1 (two postures) may therefore explain the relatively smaller effect of hand position on the JSE found in Experiments 2 and 3 than in Experiment 1. The study of Schultheis and Carlson (2013) also showed that a disappearance of the hand posture effect might, however, not only be due to an increase in the number of trials (i.e., practice). An alternative explanation for the lack of an effect of hand position in the standard Simon task could be that two effects, an enhancement of spatial processing and an enhancement of cognitive control, might work against each other in the standard Simon task. While the former should lead to an increase of the Simon effect, the latter should lead to a decrease of the effect. This assumption would be in line with current research providing evidence for fewer involvement of cognitive control in the joint Simon task than the standard Simon task (Milanese et al., 2010; Liepelt et al., 2011b). However, this assumption is currently purely speculative and needs to be tested in future research.

The response times of the joint Simon task in Experiment 1 were longer than in Experiments 2 and 3. One reason for this finding may be that participants also performed the individual and the standard Simon task in Experiment 1, while they had more practice with the joint Simon task in Experiments 2 and 3. In Experiments 2 and 3 participants performed the joint Simon task in four different hand position combinations, whereas they had only two hand position combinations in Experiment 1. The larger amount of practice with the joint Simon task in Experiments 2 and 3 may have led to a speed up of response times. This could also be the reason for the relatively smaller effect of the hand position manipulation in Experiments 2 and 3 than in Experiment 1.

### IMPLICATIONS FOR DYADIC TASK SHARING AND THE JOINT SIMON EFFECT

The present finding of an enhanced spatial processing in the hands monitor condition of the joint Simon task cannot be explained by a difference in the spatial distance of the response buttons between hands monitor and hands knee conditions since this distance was kept constant between the hands monitor and the hands knee conditions. Experiment 3 further showed that the hand posture effect found in the previous two experiments could also be induced when a responding rubber hand replaced the human co-actor. The finding of an increased JSE in the own hands monitor condition when co-acting with a rubber hand may suggest that the enhancement of spatial response processing of near hand space could reflect a domain general process. Domain general, because the optimization of task processing comes into play when external (action) events lead to a response selection problem no matter if these events are produced by living or non-living entities. This does, however, not imply that events produced by a human co-actor may not lead to a larger response selection conflict (Müller et al., 2011; Stenzel et al., 2012, 2014) and hence a larger JSE, but that the cognitive mechanisms underlying JSEs with human and non-human co-actors may not be qualitatively different (Colzato et al., 2013). Perceived differences between task setups using human and non-human co-actors may therefore be better defined as gradual changes in the strength of information processing—here the amount of referential coding. According to the logic of the referential coding account, a gradual increase of the JSE when sharing a Simon task with a human co-actor is the consequence of the increased similarity between own and others action events making the action selection conflict more difficult. The present findings may provide one way how attention and intentional weighting may interact in the joint Simon task, so that a stronger weighting of discriminative action features can counteract the effect of enhanced spatial processing of the space near one’s own hand. This conclusion is supported by the findings of Experiment 2 showing a stronger effect of hand position on the JSE when the other person’s hand was also located on the monitor, than when it was located on the knee.

The distribution analyses of Experiment 1 showing a clear functional dissociation between the joint Simon effect and the standard Simon effect also supports the referential coding account for the joint Simon effect. This finding is not in line with the core assumption of the action corepresentation account holding that individuals sharing a Simon task functionally represent the other person’s task in the same way as when performing both parts of the task alone (Sebanz et al., 2003; Vesper et al., 2010). However, when removing the functional equivalence assumption of the action corepresentation account, only assuming that own (produced) and others (perceived) action events are represented in a common code (Prinz, 1990, 1997), one can say that we experience others in the same format in which we experience ourselves—in terms of events (proximal, distal, or virtual) that we, or others produce (Jordan, 2003, 2009, 2013). In this sense, a differential coding of one’s own action in relation to another system’s action effect (human or non-human), is simultaneously referential coding as well as a social representation of intentional relations between agents through the act of distinguishing events of self and other (Jordan, 2003). The question if these processes are considered to be social or non-social may be more a matter of belief than of experimental evidence. While the former would assume an inherently social origin of common coding that generalizes also to non-human systems, the latter adopts a more individualistic and domain general perspective of the cognitive system, assuming that up to a certain level similar processes may be involved in social and non-social contexts. Independently, which perspective one takes, the main message that should come out here is that up to the given level of investigation that we achieve with the joint Simon task, the same functional mechanisms seem to be involved in processing events of human and non-human agents and differences between these two may be defined in gradual terms.

Sun and Thomas (2013) recently showed that sitting next to a friend who places his/her hand passively next to a target location does not lead to altered vision near the friend’s hand. However, after performing a joint wax-sawing task this study provided evidence for altered vision near the friend’s hand in a visual attention task. Different from that, participants in the present study were real co-actors always performing their part of the shared task together with their co-actor. This suggests that it may be a fundamental difference for attention allocation if two persons are actually sharing a task (joint action) or if they are just passively sitting side-by-side holding their hands in near task space.

A potential limitation of the present findings is that a rubber hand may activate the same bimodal neurons as a real human hand, which are currently discussed as the underlying neural source for enhanced spatial processing of the area near the hands (Graziano and Gross, 1995; Maravita and Iriki, 2004; Reed et al., 2006). Even though a rubber hand is clearly non-living, future studies will have to show if similar attention allocation processes are gained when other distracting events that are not task-related or do not have the appearance of a human body part also produce an enhancement of spatial attention to near hand space. The present design does not allow answering the question if there is something special about alternative hands or if similar attention allocation processes could be gained with other distracting events replacing the hand. However, one should be aware that even if other attention capturing stimuli, which are located on the monitor, would bias the spatial distribution of attention to one’s own hand, there is still something special about own hand position, which supports the original idea of altered vision near the (own) hand position (Reed et al., 2006; Abrams et al., 2008; Davoli et al., 2010).

Taken together, the present findings suggest that acting with the hands on the monitor lead to an enhanced spatial processing for (or biases the distribution of attention to) the area near the hands when external action events are produced in spatial reference to the actor. The enhanced spatial processing increases the action selection conflict between internally and externally activated events triggering compensatory weighting (of discriminative action features) increasing the JSE. The present study is the first to show how attention and intention may interact in the joint Simon task.

## AUTHOR CONTRIBUTIONS

Roman Liepelt contributed to the conception and design of the work. Roman Liepelt analyzed the data. Roman Liepelt contributed to the interpretation of the work. Roman Liepelt wrote the initial and final draft of the manuscript. Roman Liepelt approved the final version of the manuscript and was fully accountable for all aspects of the work.

## Conflict of Interest Statement

The author declares that the research was conducted in the absence of any commercial or financial relationships that could be construed as a potential conflict of interest.

## Footnotes

1 The Simon effect in the visual standard Simon task is typically found increase in size with shortened RTs (see e.g., Hommel, 1993; Liepelt et al., 2011b). RTs in the joint Simon task were overall faster than in the individual Simon task and the standard Simon task condition. To rule out that the significant three-way interaction of setting × hand position × compatibility in Experiment 1 (i.e., the stronger increase of the Simon effect for hands monitor than in hands knee conditions in the joint Simon task as compared to the individual Simon task and the standard Simon task) is due to faster RTs in the joint Simon task as compared to the individual and the standard Simon task, a distributional analysis was performed. RT distributions were computed for each subject and for each cell combination of setting, hand position, and compatibility. The distributions were divided into quartiles (bins) from the fastest to the slowest (see e.g., De Jong et al., 1994). Then the data were analyzed with a 3 × 2 × 2 × 4 repeated measures ANOVA with the within-subjects factors setting (individual Simon task, joint Simon task, standard Simon task), hand position (hands monitor, hands knee), compatibility (compatible, incompatible) and quartile. Most importantly, there was no significant four-way interaction of setting × hand position × compatibility × quartile, (*F*< 1) ruling out that the joint hand posture effect (i.e., the significant three-way interaction of setting × hand position × compatibility) found in Experiment 1 was due to overall faster RTs in the joint Simon task, as compared to the individual Simon task and the standard Simon task. In addition to the findings already reported in the overall RT analysis, there was a significant main effect of quartile, *F*(3,69) = 557.01, *p*< 0.001, partial η^2^ = 0.73, showing that RTs varied across quartiles. Further, there was a significant interaction of setting × quartile, *F*(6,138) = 33.57, *p*< 0.001, partial η^2^ = 0.59, showing that RTs varied the most in the standard Simon task. An interesting result was also the finding of a significant interaction of compatibility × quartile, *F*(3,69) = 10.67, *p*< 0.001, partial η^2^ = 0.72, and of a significant three-way interaction of setting × compatibility × quartile, *F*(6,138) = 14.18, *p*< 0.001, partial η^2^ = 0.38 showing not only that the size of the compatibility effects varied across quartiles, but also that the way how compatibility effects varied between joint, individual, and standard Simon tasks differed. While there was the typical increase of the compatibility effect with shortened RTs, *F*(3,69) = 26.25, *p*< 0.001, partial η^2^ = 0.53, in the standard Simon task (Hommel, 1993; Liepelt et al., 2011b), the joint Simon task showed a reversed pattern, namely a significant increase of the compatibility effect with longer RTs, *F*(3,69) = 2.81, *p*< 0.05, partial η^2^ = 0.11, indicating a functional difference of the mechanisms underlying the joint Simon effect and the standard Simon effect. Response speed did not affect the size of the Simon effect in the individual Simon task (*F* < 1). The variation of the size of the compatibility effect by hand position was not affected by response speed in all three-task settings (the joint Simon task, the individual Simon task, and the standard Simon task, all *F*s < 1).
